# Qualitative modelling of the interplay of inflammatory status and butyrate in the human gut: a hypotheses about robust bi-stability

**DOI:** 10.1186/s12918-018-0667-6

**Published:** 2018-12-17

**Authors:** Gunter Neumann, Rebecca Wall, Ignacio Rangel, Tatiana M. Marques, Dirk Repsilber

**Affiliations:** School of Medical Health (MV), Örebro University, Örebro, 70182 Sweden

**Keywords:** Gut microbiome, Dysbiosis, Bi-stability, Inflammation, Short chain fatty acids, Butyrate, Dynamical model

## Abstract

**Background:**

Gut microbiota interacts with the human gut in multiple ways. Microbiota composition is altered in inflamed gut conditions. Likewise, certain microbial fermentation products as well as the lipopolysaccharides of the outer membrane are examples of microbial products with opposing influences on gut epithelium inflammation status. This system of intricate interactions is known to play a core role in human gut inflammatory diseases. Here, we present and analyse a simplified model of bidirectional interaction between the microbiota and the host: in focus is butyrate as an example for a bacterial fermentation product with anti-inflammatory properties.

**Results:**

We build a dynamical model based on an existing model of inflammatory regulation in gut epithelial cells. Our model introduces both butyrate as a bacterial product which counteracts inflammation, as well as bacterial LPS as a pro-inflammatory bacterial product. Moreover, we propose an extension of this model that also includes a feedback interaction towards bacterial composition. The analysis of these dynamical models shows robust bi-stability driven by butyrate concentrations in the gut. The extended model hints towards a further possible enforcement of the observed bi-stability via alteration of gut bacterial composition. A theoretical perspective on the stability of the described switch-like character is discussed.

**Conclusions:**

Interpreting the results of this qualitative model allows formulating hypotheses about the switch-like character of inflammatory regulation in the gut epithelium, involving bacterial products as constitutive parts of the system. We also speculate about possible explanations for observed bimodal distributions in bacterial compositions in the human gut. The switch-like behaviour of the system proved to be mostly independent of parameter choices. Further implications of the qualitative character of our modeling approach for the robustness of the proposed hypotheses are discussed, as well as the pronounced role of butyrate compared to other inflammatory regulators, especially LPS, NF- *κ*B and cytokines.

**Electronic supplementary material:**

The online version of this article (10.1186/s12918-018-0667-6) contains supplementary material, which is available to authorized users.

## Background

The human gut harbours a plethora of microbiota able to digest and process a vast amount of dietary compounds. These are needed for the human metabolism and have regulatory implications for the immune system [15, 25]. Dysbiosis is defined as a drastic change in the composition of the gut microbiota, and is often associated with disease: major examples are Irritable Bowel Syndrome (IBS) and Inflammatory Bowel Disease (IBD) [27, 28], metabolic syndrome and type-2 diabetes [31, 54], obesity and gut problems in the elderly [7, 36]. Generally speaking, dysbiosis has been hypothesized to involve abnormal inflammatory status or at least long or even chronic, systemic low-grade inflammation [6].

Bacterial products are known to play decisive roles in triggering or down-regulating host inflammatory responses. For example, lipopolysaccharides (LPS) which are part of certain bacteria’s cell walls, are potent pro-inflammatory stimuli. Short-chain fatty acids (SCFA), as another example, are key metabolic products from fibre processing bacteria in the gut lumen [43, 53]. They have multiple effects for human health [5, 26]. Butyrate, in particular, plays a major role in the regulation of inflammation as an anti-inflammatory signal for the epithelial cells of the large intestine [8]. It is derived from microbial fermentation of dietary fibres in the colon. Anti-carcinogenic, anti-inflammatory, and barrier-protective activity in the distal gut are among many of its health-promoting effects [19, 40]. Butyrate also accounts for ≥ 70% of the energy used by healthy colonocytes [32], and it also functions as a histone deacetylase inhibitor inside the nucleus to epigenetically regulate gene expression and cell fate [10, 19]. Given its impact on a variety of mechanisms, there is a growing interest in butyrate and the microbes that produce this compound. Indeed, butyrate-producing bacteria are important for a healthy colon and, if reduced, contribute to emerging diseases such as IBD [55] and IBS [41]. For example, butyrate has been shown to down-regulate inflammation responses in Crohn’s disease through inhibition of NF- *κ*B activation in immune cells [47]. The NF- *κ*B-signalling system is implicated in the regulation of a variety of genes during immune and inflammatory responses, including those encoding pro-inflammatory cytokines such as TNF, IL-1 and IL-6. Low concentrations of butyrate have also been detected in IBD patients [52]. Treatment with butyrate enemas has been shown to reduce inflammation in this patient group [45]. Inflammatory signalling in healthy volunteers has also been shown to be affected by butyrate concentrations [20]. In addition, butyrate is believed to play a role in maintaining intestinal barrier function, since a deficit in butyrate causes tight junction lesions [40], and it decreases the permeability in intestinal cell models [38]. IBS is an example of a disease characterized by a systemic low-grade inflammation. IBS patients have also been shown to suffer from increased intestinal permeability, suggesting a deteriorated intestinal barrier function, also known as leaky gut [39, 58]. Increased intestinal permeability would allow translocation of endotoxic compounds, such as LPS, that trigger a sustained immune response. These processes are proposed to subsequently result in a low-grade systemic inflammatory response [27].

Inflammation is proposed to mediate a decreased butyrate uptake into gut epithelial cells. The specific carrier-mediated transport systems involved in the transport of butyrate from the colonic lumen into colonic epithelial cells are mono-carboxylate transporter 1 (MCT1) and sodium-coupled mono-carboxylate transporter 1 (SMCT1) [17, 44]. These transporters are down-regulated in patients with IBD [42, 49]. The reduced expression of MCT1 and SMCT1 in IBD has been suggested to be a consequence of intestinal inflammation, since treatment of human intestinal epithelial cells with pro-inflammatory cytokines (e.g., IFN- *γ* and TNF- *α*) down-regulates MCT1 expression, leading to butyrate deficiency [50].

Dysbiosis of the gut microbiota has been frequently related to reduced bacterial diversity [24]. In addition, bimodal distributions for certain phylogenetic subgroups of gut bacteria have been previously described [30], yielding the hypothesis that these species are decisive indicators of malfunction in the gut microbiota’s ecosystem. An additional level of complexity relates to the mucin layer in the large intestine, where the microbiota is mainly involved in fibre processing [53]. Mucus layer status has been proposed to determine distinct gut microbiota ecosystems called enterotypes [1, 13].

Here, we introduce and analyse a drastically simplified, qualitative model for the intricate interplay between bacteria, their products and gut inflammatory status. The key components of this dynamical model are the interactions between bacterial SCFA production, where butyrate is a key player, LPS as pro-inflammatory signal, intracellular uptake of butyrate, barrier function and inflammatory status of the gut epithelial cell layer, represented by NF- *κ*B and its regulators. We implement two models, a core model and an extended version, as systems of ordinary differential equations which are then investigated by fixed-point analyses. Our modelling approach aims at discovering general systems characteristics.

## Results

### Simplified inflammation model by Yde et al. [57]

We base our investigation on the simplistic inflammation model by [57]. It is a system with four interacting elements which will be summarized by the following symbols and their corresponding classes of agents in the inflammatory regulatory system:

N indicating pro-inflammatory elements such as NF- *κ*B or upstream in the regulatory system
R representing repressors, such as IkB, IkBe, A20, Cesanne
T representing pro-inflammatory cytokines, such as TNF, IL-1, IL-6
L equals S in the original model formulation by [57], which is an activator of T. In their model this corresponds to the bacterial load, which in turn is represented by LPS, a pro-inflammatory outer membrane compound.

Using these species (N,R,T,L), our model is based on the approach proposed by [57] with some adapted parameters, as defined in the following system of differential equations:

1 $$ \dot{N} = k_{a} \ \frac{T^{3}}{T^{3}+k_{s}^{3}} \ (1-N) - k_{ai} \ R \ \frac{N}{N+k_{r}}   $$

2 $$ \dot{R} = k_{b} \ N - k_{br} \ R   $$

3 $$ \dot{T} = k_{p} \ \frac{N^{2}}{N^{2}+k_{n}^{2}} - k_{t} \ T + k_{tl} \ L   $$

4 $$ \dot{L} = k_{bl} - k_{l} \ L   $$

Equations (1-4) represent the model by [57] with some simplifications: Diffusion is not considered, as we neglect the spatial aspect. Self-limitation is added for all elements to prevent unrealistic negative values. As pointed out by [57], both [48] and [12] already analysed the NF- *κ*B inflammatory system with respect to limit boundaries and activation threshold points: These are adequately modelled in this model, based on a Hill-function. The model in its non-spatial version is bistable, with one fixed point in the origin and another fixed point with all components (N,T,R,L) strictly positive [57].

### Core model implementation and analysis

#### Implementation of the core model

The simplified inflammation model by [57] is now further developed, and integrated with the basic interactions of butyrate, to constitute our core model. The first step is to assemble a list of interactions between elements of the inflammatory system and butyrate, in gut epithelial cells. The precise implementation of the interactions between certain elements of the model, such as butyrate, LPS, or parts of the inflammatory system, is to be understood as a coarse-grained, simplified approach to the underlying regulatory processes. Based on the biological knowledge as reviewed in the “Background” section, the relevant interactions for our proposed core model are defined: Butyrate ⊣ leaky barrier Butyrate is supposed to strengthen the intestinal barrier and thus in our model it blocks LPS influx from the gut lumen into epithelial cells. Butyrate ⊣ NF- *κ*B Butyrate blocks the process of NF- *κ*B entering the nucleus during ongoing inflammation. Inflammation (cytokines) ⊣ butyrate transport In the inflamed state, butyrate transporter proteins (as e.g. MCT1) are down-regulated.

**Fig. 1:**
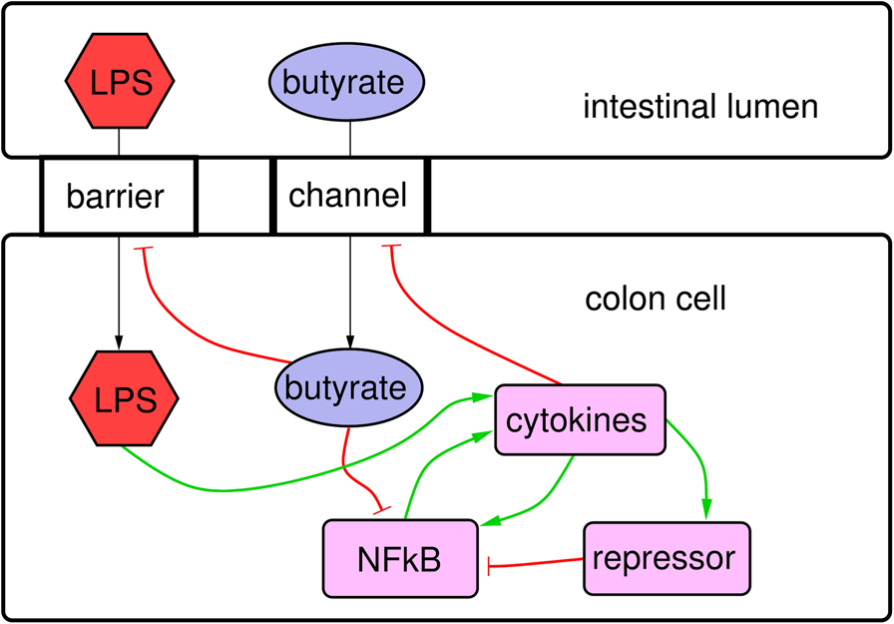
Interaction graph of the core model. LPS (L) in the intestinal lumen environment enters the epithelial cell through leaky barrier, butyrate (B) via transporter channel. Butyrate enhances barrier function of epithelial cells and blocks LPS to enter. Transport of lumen butyrate into the cell is blocked by cytokines (T). L, T, NF- *κ*B (N) and repressors (R) are representing the inflammatory model variables in the model by [57] which interact with butyrate (B), as described above and by B blocking NF- *κ*B’s transport into the nucleus (modelled as blocking NF- *κ*B). Black arrows represent transport, green arrows activation, red arrows inhibition

From these interactions, together with the non-spatial part of the simplified model of inflammation by [57], the core model is now built: Butyrate B interacts with and reduces the influx of LPS L, by strengthening the barrier function of the gut epithelial cells. Therefore, it reduces the activation of the cytokines T, as influx of LPS L is reduced. Butyrate also blocks the entrance of NF- *κ*B into the nucleus. Cytokines T on the other hand reduce the expression of the transporter for butyrate, hence diminishing its transport into the epithelial cells from the lumen. All interactions of the model by [57] together with the new interactions *via* butyrate can be collected in an interaction graph, see Fig. 1. The core model is now constructed as a five-dimensional ODE system:

LPS (*k*_*bl*_) and butyrate in the lumen (*B*_*out*_=*k*_*Bo*_) concentrations are modelled as linear diffusion parameters since they are assumed constant. Also NF- *κ*B (*N*) is recruited from the cytosol by the term (*N*_0_−*N*) which we normalize as in [57] to (1−*N*) in Eq. 5. Butyrate (*B*) acts as an inhibitor for the NF- *κ*B transport into the nucleus, by a linear term *k*_*bn*_ (see Eq. 5). NF- *κ*B is activated through cytokines and itself activating cytokines in turn. Butyrate in the epithelial cell can be provided by the reservoir (*k*_*Bo*_) in the gut lumen, modelled as a linear diffusion process with parameter *k*_*d*_, or by a transporter (first term on r.h.s. of Eq. 9), with a reduction caused by L via T, with the parameters *k*_*tl*_ (Eq. 7, last term r.h.s.) and *k*_*bt*_ (Eq. 9, first term r.h.s.). The influx of L will be reduced by the blocking effect of butyrate on the leaky barrier with parameter *k*_*lb*_ (Eq. 8, first term r.h.s.). *k*_*B*_ is a linear degradation rate for butyrate in the lumen, which we assume to be constant over time. For the inhibition of T on B (inflammatory negative effect on butyrate transport) a Hill function of second order is used (see Eq. 9, first term r.h.s.), as this allows to consider possible cooperative effects. The same consideration holds for Eq. 1.

This is now our core model:

5 $$ {}\dot{N} = k_{a} \ \frac{T^{3}}{T^{3}+k_{s}^{3}} \ (1-N) - (k_{ai} \ R + k_{bn} \ B) \ \frac{N}{N+k_{r}}   $$

6 $$ \dot{R} = k_{b} \ N - k_{br} \ R   $$

7 $$ \dot{T} = k_{p} \ \frac{N^{2}}{N^{2}+k_{n}^{2}} - k_{t} \ T + k_{tl} \ L   $$

8 $$ \dot{L} = k_{bl} \ \frac{k_{lb}^{2}}{B^{2}+ k_{lb}^{2}} - k_{l} \ L   $$

9 $$ \dot{B} = k_{Bo} \ \frac{k_{bt}^{2}}{T^{2}+k_{bt}^{2}} + k_{d} \ k_{Bo} - k_{B} \ B   $$

In Eqs. 5 to 9, the ODE system for the respective components in Fig. 1 is defined. While Eqs. 5 to 8 reflect essentially the model by [57], Eq. 9 represents its extension with butyrate-related interactions. In Eq. 5, a limitation factor in the last two terms was added, to avoid negative values of *N*. According to [33], in Eq. 9, a linear dependency upon the butyrate influx to the epithelial cell was assumed, as mediated by the transporter (e.g. MCT1; first term r.h.s.) and also by the diffusion through the epithelial cell (second term r.h.s.). This is due to enhanced expression of transporter proteins, as a regulatory effect by butyrate itself [33]. The model parameters are partly already reduced in number to avoid unnecessary redundancy. Scaling of variables is not considered here, since the model allows every absolute value for the variables in general, except for *N*.

#### Fixed point analysis of the core model

**Fig. 2:**
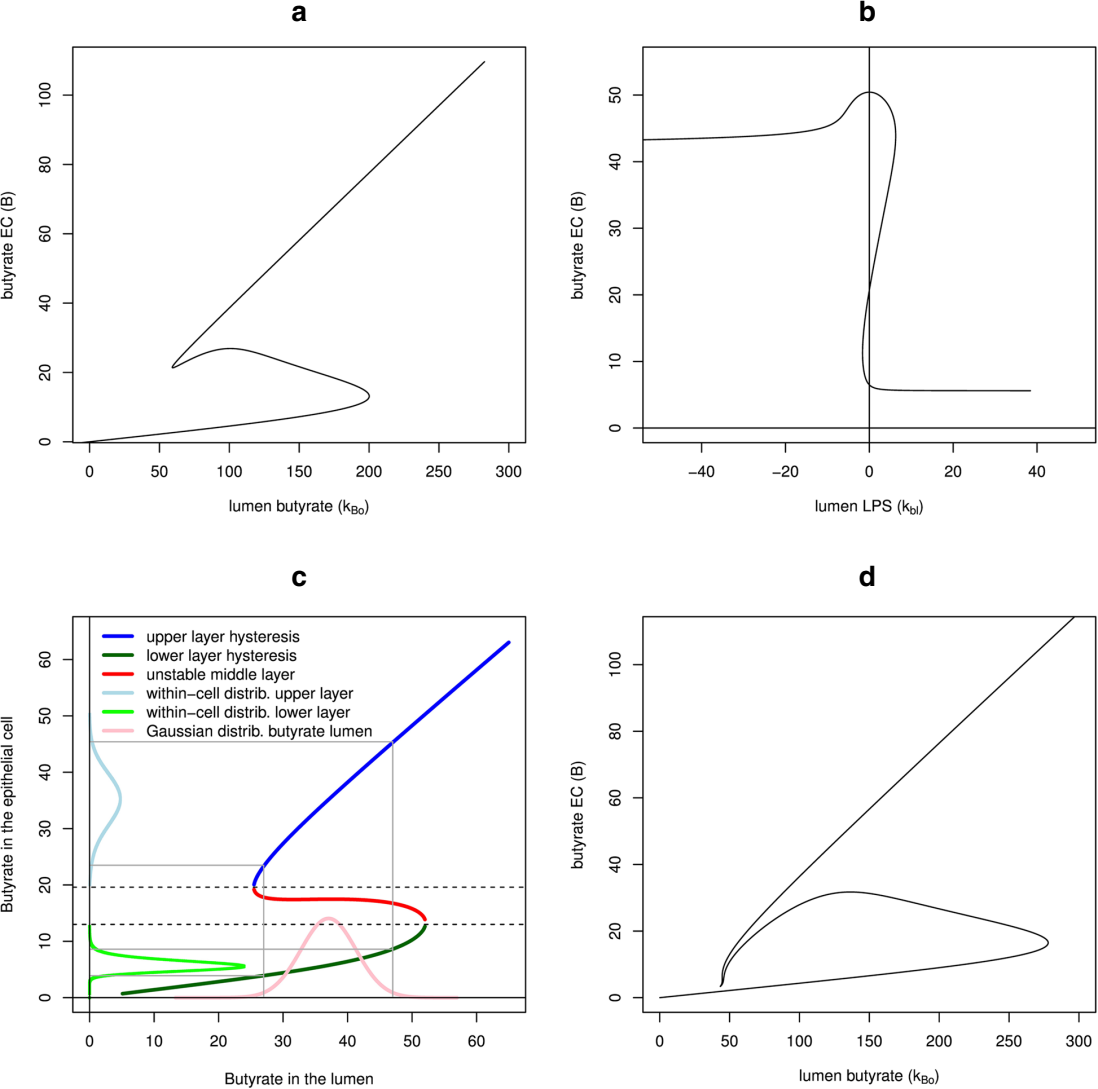
Hysteresis curves as evoked by parameters butyrate in the lumen or LPS in the lumen. **a** Hysteresis driven by lumen butyrate, as observed for the core model. Abscissa: butyrate in the gut lumen *k*_*Bo*_, ordinate: butyrate in the epithelial cell *B*. The hysteresis curve is a plot of all fixed points, i.e. no further change in concentrations: $\dot {N}=\dot {R}=\dot {T}=\dot {L}=\dot {B}=0$, of Eqs. 5 to 9, for each value of *k*_*Bo*_. Parameters: *k*_*a*_=12,*k*_*s*_=1.0,*k*_*ai*_=5,*k*_*r*_=0.5,*k*_*bn*_=4.7,*k*_*b*_=5,*k*_*br*_=0.5,*k*_*n*_=0.2,*k*_*p*_=7,*k*_*t*_=0.2,*k*_*tl*_=2.8,*k*_*bl*_=1.02,*k*_*lb*_=3.4,*k*_*l*_=0.7,*k*_*bt*_=2.1,*k*_*d*_=0.125,*k*_*B*_=2.9. The limit points on the hysteresis of the core model have normal form coefficients *a* left/right: *a*=−0.1079160/−0.01114689. **b** Hysteresis driven by lumen LPS *k*_*bl*_, as observed for the core model. Abscissa: LPS in the gut lumen *k*_*bl*_, ordinate: butyrate in the epithelial cell *B*. Parameters as for (a), but with fixed *k*_*Bo*_=130. **c** Schematic view of the transformation of the butyrate distribution in the lumen over the hysteresis to the bimodal distribution of within-cell butyrate, as depicted on the y-axis. **d** Hysteresis observed for the extended model. This model includes a positive feedback loop between butyrate in the epithelial ⇔ mucin production ⇔ SCFA producing microbiota. The area of bi-stability is broadened for this model

A fixed point analysis of our core model, as defined in Eqs. 5 to 9, was conducted. From Fig. 2a, *k*_*Bo*_ appears as a suitable parameter for the switch between inflamed state (*B* low) and healthy state (*B* high). Around the average value (here *k*_*Bo*_≈130) a bistable system exists. There are three fixed points of which the upper and lower fixed points are stable. The middle fixed point separates both the upper and lower layer of the hysteresis, and hence is unstable. Below a threshold (*k*_*Bo*_≈ 55) the system changes to a low concentration of butyrate within the epithelial cell (*B* low; inflamed state), and above a threshold (*k*_*Bo*_≈ 200) the healthy within-cell concentration can be recovered (non-inflamed state).

**Fig. 3:**
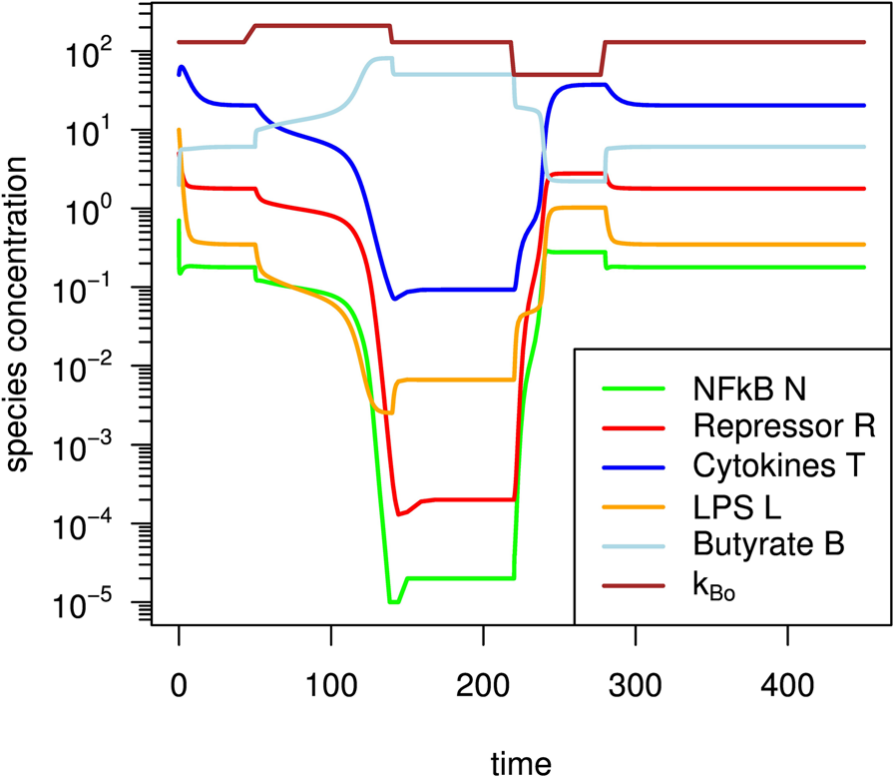
Bistable dynamics of the core model as driven by lumen butyrate. Pulsing of the butyrate concentration in the lumen *k*_*Bo*_ around an average value and its influence on the switch from the inflamed state to the healthy state. The brown line represents the concentration of butyrate in the lumen *k*_*Bo*_ and N (green), R (red), T (blue), L (yellow), B (orange) lines, respectively

Figure 3 shows the model dynamics as driven by pulsed changes of the butyrate concentration in the intestinal lumen (*k*_*Bo*_). Here, driving means that a change in the investigated parameter can result in the switching of the bistable system from one of its stable fixed points to the other. Such a change can be observed from one fixed point of the steady state system to the other, mediated by *k*_*Bo*_-pulses in both directions (brown line). *B*_*out*_=*k*_*Bo*_ can trigger the switch between both fixed points around *B*_*out*_=130. Above a threshold (*k*_*Bo*_=200 between time =50-170) the non-inflamed state is triggered, leading to a significant reduction of the variables N, R, T, and L. Below a threshold (*k*_*Bo*_=50 with time = 200 - 270) the system switches back to an inflamed state, where N, R, T and L are established on higher values again. The following special fixed points were found: Inflamed state fixed point at *N* =0.178369, *R* = 1.783690, *T* =20.365623, *L* = 0.347144, *B* =6.079745; non-inflamed state fixed point at *N* =0.00002003, *R* = 0.00020025, *T* = 0.09248014, *L* = 0.00660570, *B* =50.382992.

A constant average level of butyrate supply (by bacterial fermentation processes) is assumed available in the lumen, for the state space region of bi-stability of the core model. In such a situation, butyrate concentration in the intestinal lumen (*k*_*Bo*_) is a proper parameter for a switch between both observed fixed points around an intermediate “normal” operating mode.

We also investigated if lumen LPS, *k*_*bl*_, could act as driver of the core model’s bistability. This however is only partly the case, as shown in Fig. 2b. When starting with high levels of within-cell butyrate, increasing levels of lumen LPS lead to a switch of the system to an inflamed state, with small within-cell butyrate values. However, there is no possibility to use decreasing lumen LPS values to re-establish higher within-cell butyrate values and a non-inflamed state again (as the necessary negative values for this parameter are not biologically defined). For these analyses, lumen butyrate was kept stable at *k*_*Bo*_=130.

The qualitative bistable behaviour of the core model (5 to 9) is stable under variations of parameters in general, as shown in more detail below. The boundary of the N,R,T,L,B-system is repulsive due to self-limitation so that the hysteresis is generally attracting. Infinity is repulsive and the origin is repulsive such that at least one finite equilibrium always exists. To obtain more realistic relations between inflamed state (NF- *κ*B concentration high) and non-inflamed state (NF- *κ*B concentration low), the experimental results from [3] were used, with a ratio of inflamed/non-inflamed NF- *κ*B levels in the nucleus of ≈100:1. To obtain comparable values in the core model, parameter choices were adapted for the model part based on the work by [57].

Additional file 1: Figures S1 A and S1 B show how NF- *κ*B (N) or the cytokines (T) are effected by lumen butyrate driven hysteresis respectively.

#### Transformation of an average Gaussian distribution with the hysteresis results in bi-modality

In Fig. 2c it is exemplified how the observed hysteresis in our model could be used to transform an average Gaussian distribution of lumen butyrate levels into a bi-modal distribution of within-cell butyrate levels: Starting with the hysteresis from model 5 to 9, with the three layers (upper (blue)- middle (red) and lower (green)), note that the middle layer is unstable whereas the upper and lower layers are stable. We assume a Gaussian distribution of the butyrate distribution (p(x), pink curve) in the average gut lumen. The transformation of p(x) over the upper and lower layer of the hysteresis *y*=*h*(*x*) to the new distribution of butyrate in the epithelial cell, q(y), with Eq. 10, gives for each layer of the hysteresis a single peak in the corresponding colour (lightblue for the upper layer, lightgreen for the lower layer). Both transformed distributions (lightblue and lightgreen) constitute the overall bi-modal distribution of the butyrate concentration in the epithelial cell. Grey horizontal and vertical lines show corresponding sections for the transformation process. It is assumed that upper and lower layers occur with the same probability. A proper fitting of the curve would have to rely on fitting the probability for the upper or lower stable layer using experimental data.

Transforming a probability distribution *p*(*x*) by a function *h*(*x*)=*y* we get a new distribution *q*(*y*) by:

10 $$ q(y) = \frac{p(x)}{h'(x)} \, \ \ \ where \ \ y=h(x).   $$

Here we can set *h*(*x*) as the hysteresis, *p*(*x*) as the Gaussian distribution. Since the hysteresis is not everywhere unique we have to split the transformation for the upper layer and lower layer and weight each of them by an appropriate factor if necessary. Furthermore, the hysteresis has two points *x*_1_,*x*_2_ where *h*^′^(*x*_*i*_) (*i*∈{1,2}) tends to infinity. These points are biologically not stable since a jump towards the stable layer is more likely to happen than a fixation at this point. These points can therefore be neglected for our computations.

Consequences for the predicted bi-modal within-cell butyrate distribution in a population become first evident when considering our proposed extended model now in the following section.

### Extended model implementation and analysis

An extension of the core model is proposed, with feedback relations describing interactions between mucin and butyrate-producing bacteria, which colonise the mucus layer of the gut epithelium. The fermentation products of SCFA-producers, among them butyrate, are indispensable for functioning of gut epithelial cells. The gut epithelium produces a mucin layer. This mucin layer is the place where many SCFA-producing bacteria colonise [14, 22]. Here are the three additional interactions: butyrate → mucin layer High levels of butyrate in the epithelial cells promote mucin layer production [2, 14]. mucin layer → SCFA producers A more pronounced mucin layer promotes colonisation by SCFA producers. SCFA producers → butyrate SCFA-producing bacteria produce lumen butyrate.

These three additional interactions constitute a positive feedback-loop for butyrate inside the epithelial cells. As there is a two-step relation (butyrate → mucin → mucin-adhered bacteria), an extra quadratic term with necessary self-limitation is used. The quadratic term is justified by the double feedback (mucin & microbiota) and the self-limitation due to crowding limitation. For implementation of these additional interactions, a modified version of Eq. 9 is proposed as follows:

11 $$\begin{array}{@{}rcl@{}} \dot{B} = & k_{Bo} \ \frac{k_{bt}^{2}}{T^{2}+k_{bt}^{2}} \cdot \frac{B^{2}}{\left(B^{2}+k_{2B}^{2}\right)} + k_{d} \ k_{Bo} - k_{B} \ B  \end{array} $$

Here, *k*_2*B*_ represents the threshold for the Hill curve. This extra term also models the fact that at zero level of butyrate there is no recovering of the butyrate system, which is neglected here, since a completely zero level is not biologically reasonable, and the origin is repulsive.

Fixed-point analysis results for the proposed extended model turned out similar to those of the core model. However, Fig. 2d demonstrates an increased separation of the bistable layers due to the additional feedback.

Considering the transformation of a gut lumen Gaussian distribution of butyrate towards a bimodal within-cell distribution within a population, these findings can now be interpreted with regard to the proposed extended model: a negative correlation between inflammation and the thickness of the mucin layer is hypothesized. The higher the inflammation markers, the thinner the mucin layer and vice versa. This corresponds to the initially defined interaction – a positive correlation – between within-cell butyrate and the mucin layer [2, 14]. Consequently, a bi-modal distribution of within-cell butyrate can be hypothesized to result in a bi-modal distribution of mucin layer thicknesses, and, thus, could result in a bi-modal distribution of SCFA-producing bacteria, within a population.

### In depth mathematical analyses of the core model

#### Existence of interior fixed points and stability of the hysteresis

In this section, we elucidate the more theoretical background of our statement about robust hysteresis, and thereby bistability, in the investigated system (here: the core model). We show that at least one fixed point exists in the positive orthant *N*,*L*,*R*,*T*,*B*≥0, in Fig. 2a, Eqs. 5 - 9. We denote the maximum and minimum of all constants *k*_∗_ from Eqs. 5 to 9 by:

12 $$\begin{array}{@{}rcl@{}} u: \ &=&\ \max{k_{\ast} \ >0,} \end{array} $$

13 $$\begin{array}{@{}rcl@{}} d: \ &=& \ \min{k_{\ast}}\ >0. \end{array} $$

For lumen butyrate concentration, we assume that *k*_*Bo*_>0. Then, the following limit relations for *N*,*L*,*R*,*T*,*B*>0 can be observed:

1. N:
  - case *N*→1: $\Rightarrow \dot {N} = - \left (k_{ai} \ R + k_{bn} \ B\right) < 0$
  - case *N*→0: $\Rightarrow \dot {N} = k_{a} \ \frac {T^{3}}{T^{3}+k_{s}^{3}} > 0$
2. R:
  - case *R*→*∞*: $\Rightarrow \dot {R} \sim - k_{br} \ R < 0$
  - case *R*→0: $\Rightarrow \dot {R} = k_{b} \ N > 0$
3. T:
  - case *T*→*∞*: $\Rightarrow \dot {T} \sim - k_{t} \ T < 0$
  - case *T*→0: $\Rightarrow \dot {T} = k_{p} \ \frac {N^{2}}{N^{2}+k_{n}^{2}} + k_{tl} \ L > 0$
4. L:
  - case $L > \frac {k_{bl}}{k_{l}} $$\Rightarrow \dot {L} < 0$
  - case *L*→0: $\Rightarrow \dot {L} = k_{bl} \frac {k_{lb}^{2}}{B^{2}+ k_{lb}^{2}} > 0$
5. B:
  - case *B*→*∞*: $\Rightarrow \dot {B} \sim - k_{B} \ B < 0$
  - case *B*→0: $\Rightarrow \dot {B} = k_{Bo} \ \frac {k_{bt}^{2}}{T^{2}+k_{bt}^{2}} + k_{d} \ k_{Bo} > 0$

We construct a box *P*, which is mapped into itself under the ODE of Eqs. 5 to 9. Hence by Brouwer’s fixed point theorem [4] there exists at least one fixed point in *P*°. We show that there is no fixed point on the boundary of the box *P*. First observe that for

14 $$ N,L,R,T,B \ \leq \ \epsilon\ > \ 0 \  $$

with *ε* sufficiently small, both $\dot {L}, \dot {B} > 0$ since *d*>0. Hence, there is no fixed point in the ball *B*_*ε*_=∥(*N*,*R*,*T*,*L*,*B*)∥≤*ε* around the origin {*N*,*R*,*T*,*L*,*B*}=0. We set $L_{0}=\frac { k_{bl}}{k_{l}}+\delta $, where *δ*>0. Let *K*>*L*_0_ be large enough such that for *L*≤*L*_0_ case (a) from limit relations 2, 3 and 5 are satisfied for *R*,*T*,*B*≥*K*. Based on these assumptions, we analyse the case, where *B*,*L*≥*ε* and the different cases with *N*,*R*,*T*→0:

15 $$\begin{array}{@{}rcl@{}} T=0, & N,R >0 & \Rightarrow \dot{T} >0  \end{array} $$

16 $$\begin{array}{@{}rcl@{}} N=0, & R,T >0 & \Rightarrow \dot{N} >0 \end{array} $$

17 $$\begin{array}{@{}rcl@{}} R=0, & N,T >0 & \Rightarrow \dot{R} >0 \end{array} $$

18 $$\begin{array}{@{}rcl@{}} R,N=0, & T >0 & \Rightarrow \dot{N} >0, \dot{R} =0 \end{array} $$

19 $$\begin{array}{@{}rcl@{}} R,T=0, & N >0 & \Rightarrow \dot{T} >0, \dot{R} >0 \end{array} $$

20 $$\begin{array}{@{}rcl@{}} N,T=0, & R >0 & \Rightarrow \dot{N} =0, \dot{T} >0 \end{array} $$

21 $$\begin{array}{@{}rcl@{}} N,T,R=0, & & \Rightarrow \dot{N} =0, \dot{T} >0, \dot{R} =0  \end{array} $$

From relations 15 to 21 it can be deduced that the positive orthant *N*,*L*,*R*,*T*,*B*≥0 is invariant and that there is no fixed point on its border. Furthermore, we can now determine the box *P* by:

22 $$\begin{array}{@{}rcl@{}} N, R, T, L, B &\geq& 0  \end{array} $$

23 $$\begin{array}{@{}rcl@{}} L &\leq& L_{0}  \end{array} $$

24 $$\begin{array}{@{}rcl@{}} N &\leq& 1  \end{array} $$

25 $$\begin{array}{@{}rcl@{}} R, T, B &\leq& K \ . \end{array} $$

From Eqs. 23 to 25 and cases (a) from limit relations 1 to 5, we can see that the upper border of *P* is repulsive since the vectorfield defined by Eqs. 5 to 9 points into *P*^*o*^. Since *δ* was not bound from above, the box *P* can be arbitrarily large in the positive orthant except for *N* which is bound by definition. Hence, the previous numerically obtained bifurcation curve from Fig. 2a has at least one fixed point. In order to investigate the special form of the hysteresis we have to use a continuation program and evaluate its stability. For the parameter set of Fig. 2a, we can give the sign of the normal form coefficient *a* of the normal form approximation at the left and right limit points with active parameter *k*_*Bo*_ (values for a see Fig. 2a, as computed by matcont [29]). Both are negative (*a*<0), indicating a stable top and bottom layer of the hysteresis. There are no other bifurcation points for the hysteresis for the used parameters. For other choices of parameters, we could, in principle, also get stability changes within the hysteresis layers, indicating bifurcation points with emerging limit cycles. Brouwer’s fixed point theorem only provides the existence for one or more fixed points in the positive orthant for our model in general. From a biological perspective, these conclusions can be interpreted as statements about the robustness of bistability in the investigated system and their necessary preconditions. These preconditions are motivated by experimental evidence.

#### Limit behaviour for various parameters

As in Fig. 2a we can see that for large *k*_*Bo*_ the upper layer of the hysteresis converges to a line through the origin after being bowed away from it by the presence of inflammation for low *k*_*Ko*_ values. A short limit analysis for *k*_*Bo*_→*∞* is available through:

26 $$\begin{array}{*{20}l} {}\dot{B}= 0 & \ \Rightarrow \ B= \frac{k_{Bo}}{k_{B}}\left(\frac{k_{bt}^{2}}{T^{2}+k_{bt}^{2}} + k_{d}\right),\\ &\qquad\qquad for \ \ \ k_{Bo} \rightarrow \infty \ \Rightarrow \ B \rightarrow \infty \\ \end{array} $$

27 $$\begin{array}{*{20}l} {}\dot{L}=0 & \ \Rightarrow \ L= \frac{k_{bl} \cdot k_{lb}^{2}}{\left(B^{2}+ k_{lb}^{2}\right)\cdot k_{l}}, & for \ \ \ B \rightarrow \infty \ \ \Rightarrow L \rightarrow 0 \\ \end{array} $$

28 $$\begin{array}{*{20}l} \dot{N}=0 & \Rightarrow \dot{N} \leq k_{a} - k_{bn} B \frac{N}{N+k_{r}},& \ for \ B \rightarrow \infty \! \Rightarrow\! N \!\rightarrow \! 0  \\ \end{array} $$

29 $$\begin{array}{*{20}l} \dot{R}=0 & \ \ \Rightarrow{R} =N \frac{k_{b}}{k_{br}}, & \ \ for \ B \rightarrow \infty \ \& \ (1.8) \ \ \Rightarrow \ \ R \rightarrow 0 \\ \end{array} $$

30 $$\begin{array}{*{20}l} {}\dot{T}=0, & \ \ \ B \rightarrow \infty \ \& \ (1.7)\ \&\ (1.8) & \Rightarrow \ \dot{T} \leq 0 \ \ \Rightarrow \ \ T \rightarrow 0 \\ \end{array} $$

31 $$\begin{array}{*{20}l} {}\dot{B} = 0, &\quad k_{Bo} \rightarrow \infty \ \& \ (1.6) - (1.10)&\Rightarrow B \approx k_{Bo} \ \frac{\left(1 + k_{d}\right) }{k_{B}} \ \  \end{array} $$

For large values of lumen butyrate, *k*_*Bo*_, the upper layer of the hysteresis follows a negative linear trend, with slope $ \frac {(1 + k_{d}) }{k_{B}} $, which is larger than that at the origin with ongoing inflammation. This results in the bi-modal distribution, as explained in Fig. 2c, with different height and width as predicted by Eq. 10. Biologically, this translates again as a statement about robust bistability in our investigated system, as being rather independent of parameter choices in our modelling approach.

## Discussion

### Robust bistability from the interplay of two antagonists: inflammation and butyrate

The cross-talk between butyrate and the inflammatory state of human gut epithelium cells comprises complex interactions, including feedback between different elements, and therefore constitutes a problem eligible for a systems biological analysis. We built a core ODE dynamical model for this set of interactions based on previous knowledge and additional terms. Choice of parameters for our ODE model was based both on previous modelling work [57] and further prior knowledge, but no dynamical data, rendering all results essentially qualitative in character. Our analyses showed a clear bistable behaviour for the system, characterised by a hysteresis-like fixed point curve (Figs. 2 a, b and d). The found bistability is largely independent of the specific choices of dynamic parameters. Our model predicts that butyrate concentration in the gut (*k*_*Bo*_) is a possible driver for the switch between the two stable, non-zero fixed points in the system, a non-inflamed state with high inner butyrate levels as opposed to the inflamed state with low inner butyrate concentrations. However, other model-specific parameters can also control this switch-like behaviour, for example, lumen LPS. Essentially, the system, even in its extended form, consists of two antagonistic parts, inflammation and butyrate. Each of these parts suppresses the other, resulting in the observed robust bistability. Both lumen butyrate and lumen LPS can act as drivers of this system, determining which of the two switch-like states (the two stable non-zero fixed points) would dominate. However, only butyrate can drive the system both into the inflamed state, by decreasing lumen butyrate levels, and back into the non-inflamed state, by sufficiently increasing lumen butyrate levels, at least during a pulse.

Inflammation is modelled qualitatively, as a simplistic interplay between three species, NF- *κ*B (N), the cytokines (T), and a repressor (R) with only one non-zero stable fixed point. In our modelling approach these species are of comparatively *re-active* character, responding to, rather than driving, the switch between inflamed or non-inflamed status. However, the modelled system is analysed as if cut-out from the larger regulatory network of the organ, here, the human gut. In reality, there might be additional regulatory interactions, and certain situations in which these get strong and decisive. Other than NF- *κ*B, which is an internal cellular signal, cytokines of different kinds also act as inter-cellular signals. Such pro-inflammatory or anti-inflammatory cytokines are known to originate from other places in the body, and, by regulating the NF- *κ*B inflammatory system, also drive the system towards an inflamed (pro-inflammatory cytokines) or an uninflamed (anti-inflammtory cytokines) state. A virus or bacterial infection of the gut, for example, could lead to additional signals resulting in high NF- *κ*B levels, and hence drive the system towards inflammation. Biologics treatment of inflammation via anti-TNF antibodies, an upstream regulator of NF- *κ*B, is another example for an alternative driver in the analysed system [35]. In summary, many elements of the system have the potential to induce a bi-stable behaviour. However, our simplified model shows that lumen butyrate on its own, as well as lumen LPS, can drive a bi-stable behaviour. More data is needed to elucidate the behaviour of the system under a wide range of physiological conditions.

The proposed core model constitutes a very simplified account of the interaction between inflammation and fermentation products from gut bacteria (butyrate in particular). It cannot therefore serve to generate quantitative conclusions about concentration thresholds or time-scales. However, from our point of view, it still can provide useful insights into regulatory mechanisms of inflammation in the gut and its potential consequences. As opposed to the model in [57] that shows one non-zero fixed point, our core model has two non-zero stable fixed points (and an unstable third one). The observed bi-stability would be lost if the system was parametrised in a way such that interactions in the inflammation-regulatory subsystem (N, T, R) would be significantly damped or lost. Experimental evidence suggests that the modelled interactions are not marginal in a real scenario and, therefore, our model provides a simplistic but also realistic account of this system.

Our model also links to possible ways to interfere with the two observed antagonistic states. We refer to treatment of inflammation with butyrate enemas as a therapy (compare e.g. [51]) or appropriate dietary interventions, e.g. butyrate-promoting dietary fibres (compare e.g. [9]), especially for situations of low-grade inflammation.

### Model-based insights on the bimodality of the gut microbiota

Assuming an average Gaussian distribution of gut lumen butyrate concentrations in a collection of systems (cohort, population), our core model predicts a bimodal transformation for the epithelial cell inner butyrate levels. Reasoning about possible consequences of this characteristic of our modelled system leads over to a more speculative part of our work, discussed in the following. The proposed extended model builds on additional interactions that are hypothesized to operate in the gut-microbiota system. Experimental evidence indicates that an inflamed gut epithelium produces less of a mucus layer, and also results in a damaged mucus layer [21]. As butyrate counteracts an inflamed state in epithelial cells, it might indirectly promote a more pronounced/intact mucus layer. Butyrate is also an important energy source for gut epithelial cells [23] and might therefore indirectly increase mucin production. Important SCFA-producing bacteria are dependent on the mucus layer as their habitat [11]. Hence, these proposed interactions result in a positive feedback loop from gut lumen butyrate, via butyrate within the cell, and back, mediated by the mucus layer. This positive feedback loop would also constitute an interaction to within-cell butyrate levels, as their control of inflammation allows mucus production, and thereby influence indirectly microbiota composition in the gut lumen. Simulations in [46] showed a positive feedback between mucin producing epithelial cells and mucin feeding bacteria. This line of argument leads us to propose a possible explanation for the bimodality of gut microbiome species compositions as described in a recent work by [30]: A possible effect of the bi-stability in the intestinal inflammatory system could have consequences also for the composition of the gut microbiome. In [30] five taxonomic groups were classified as bimodal with regard to their abundance distribution. Also [18] described an essential part of mucin-related microbiota as bimodally distributed. Some of these are SCFA- and specifically butyrate-producers [11, 37, 53, 56]. Moreover, even the key species of the proposed three enterotypes by [1] correspond to the bimodal types as observed in [30].

## Conclusions and outlook

Our model shows robust bistability as a result of the feedback between two antagonists, microbial-derived butyrate and gut epithelial inflammation. These observations warrant further mathematical and experimental studies on the emergence of bistability. Additional experimental data is required to parametrise our modeling approach, and would need to involve a dynamical screening of the concentration of key components or derived variables thereof. In vitro experiments with gut epithelial cell-derived cell cultures are the basis to further understand key pathways and signals involved in the butyrate-inflammation interplay. Organoid studies on the same topic are started, as well as *ex vivo* studies involving Ussing chamber experiments [16], where barrier function can directly be related to inflammatory signals. Finally, in the clinical setting, carefully managed butyrate bolus experiments, as well as tightly controlled fibre-rich diets, are already under way. All these levels of experimental evidence are necessarily dynamic in nature as to enable systems biological analysis of mechanistic hypotheses.

## Methods

### Aim, design and software for analysis

The aim of the study is to manifest the fundamental antagonistic behaviour between inflammation of the gut epithelial cell layer and specific bacterial products in the large intestine, represented here by butyrate. We analyse the ODE-model based on [57] extended by suitable interaction terms with butyrate, as described in detail in the “Results” section, model development. First, we observe by simulations with Matlab [34] a bistable behaviour, visible as hysteresis between antagonistic variables in our mathematical model. A switch-like behaviour is modelled also by Simulink (available as add-on for Matlab) showing the operation of the hysteresis. A continuation analysis is implemented using the matcont (package for Matlab) [29], giving bifurcation points for determining the stability of the hysteresis. A plausible stability analysis for the variables is given, indicating the global stability of the top and bottom layer of the hysteresis. A probability transformation of an assumed Gaussian distribution of butyrate in the lumen, mediated by the hysteresis, results in a qualitative similarity to observed bimodal distributions within the gut intestinal microbiome as observed in [30].

## Availability of data and materials

Simulation and analysis scripts are made available as tar-archive (Additional file 2) within the section Additional files.

## Additional files

Additional file 1 **Figure S1.** Hysteresis effects driven by lumen butyrate on further variables in the core model. A: for NF- *κ*B (N). B: for cytokines (T). Parameters as for Fig. 2a. (JPG 40 kb)

Additional file 2 MATLAB scripts. tar archive with MATLAB scripts for simulating the hysteresis and for transforming a Gaussian distribution via the simulated hysteresis. (TAR 966 kb)
