# Supplementary figures and images for: Qualitative modelling of the interplay of inflammatory status and butyrate in the human gut: a hypotheses about robust bi-stability
Source: BMC Syst Biol. 2018 Dec 17;12:144. doi: 10.1186/s12918-018-0667-6 (PMC6296070; doi:10.1186/s12918-018-0667-6)

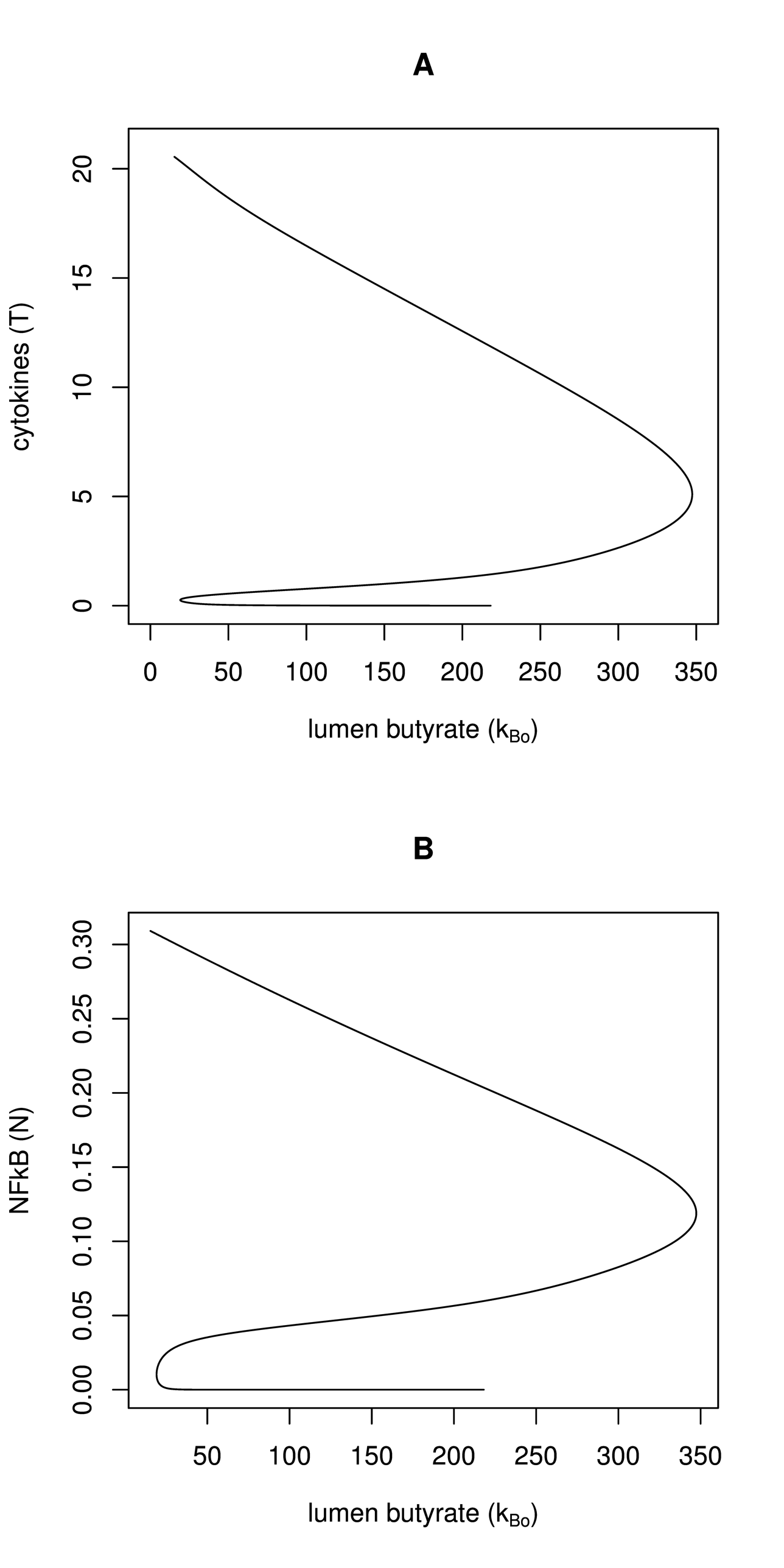

Supplement: Supplementary file 1 — Figure S1. Hysteresis effects driven by lumen butyrate on further variables in the core model. A: for NF- κB (N). B: for cytokines (T). Parameters as for Fig. 2a. (JPG 40 kb) [file 12918_2018_667_MOESM1_ESM.jpg]
